# Stem Cell and Synthetic Embryo Models: Advances, Applications, and Ethical Considerations

**DOI:** 10.1007/s12015-025-10890-z

**Published:** 2025-05-20

**Authors:** Hany E. Marei

**Affiliations:** Department of Cytology and Histology, Faculty of Veterinary Medicine, Mansoura University, Mansoura, 35116 Egypt

**Keywords:** Stem cells, Synthetic embryo models (SEMs), Pluripotent stem cells (PSCs), Embryoid bodies, Organoids, Early human development, Ethical considerations, Regulatory frameworks, Biomedical research ethics, In vitro embryogenesis, Reproductive bioethics

## Abstract

Independent traditional gametes and recent advances in stem cell biology have made it possible to create synthetic embryo models (SEMs), altering our capacity to study early human development, congenital diseases, and regenerative medicine. By recreating key developmental events in vitro, these models provide unmatched insights into embryogenesis and provide creative platforms for disease modeling, drug discovery, and individualized therapy. The quick development in SEM research raises serious ethical, legal, and regulatory questions that call for creating transparent control systems. The methods applied in SEM fabrication, their biomedical applications, and the moral issues connected with their use are investigated in this review. We also look at future directions, including enhancing ethical frameworks, adding artificial intelligence, increasing model fidelity, and encouraging public participation. Through multidisciplinary cooperation, SEMs might address these problems and transform developmental biology, advancing ethical scientific advancement.

## Introduction

Ethical and technical restrictions have made the multifarious and painstaking process of embryogenesis difficult for research. Synthetic embryo models (SEMs) generated from pluripotent stem cells (PSCs) offer a substitute for traditional embryology that lets researchers copy early development in vitro. These models help us better understand human development and can be used in therapeutic approaches and disease modeling. Finding SEMs has sparked a revolution in regenerative medicine and developmental biology. SEMs’ foundation is stem cells’ ability to self-organize and reproduce embryogenesis free of fertilization. Thanks to the pioneering work of Magdalena Zernicka-Goetz and Jacob Hanna, stem cells can now create embryo-like structures that nearly resemble early-stage embryos [1, 2]. This revolutionary technology offers new insights into uncommon diseases, genetic disorders, and tailored medication, thereby transforming biomedical research.

**Fig. 1:**
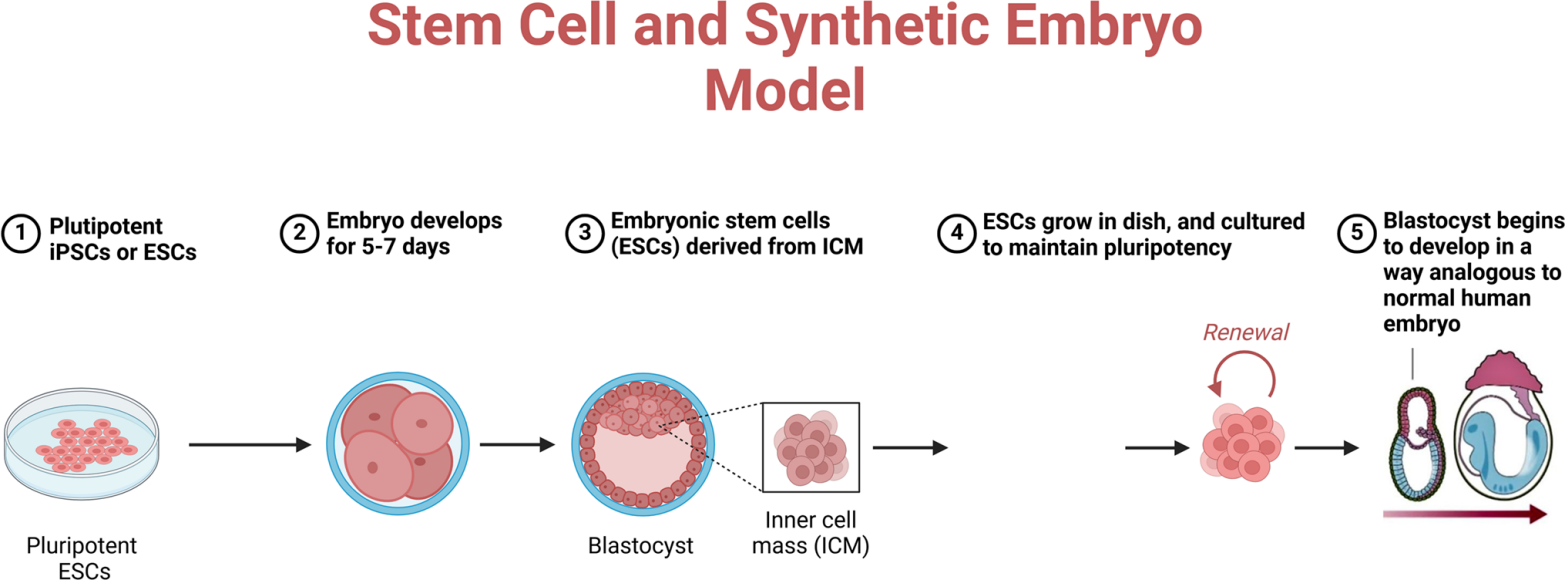
Stem cell and synthetic embryo model. Unlike regular embryos, which form from the union of gametes, SEMs are developed from pluripotent stem cells (PSCs), such as induced pluripotent stem cells (iPSCs) and embryonic stem cells (ESCs). Because of this advancement, new approaches to studying early embryogenesis can now be taken without being bound by the same ethical guidelines that govern human embryos. Several techniques have been developed to generate SEMs, including trophoblast integration, gastruloid development, blastoid creation, and self-organizing stem cell aggregation. By enabling the in vitro study of developmental processes, these methods shed light on early lineage specification, cellular differentiation, and organogenesis

Unlike traditional embryos, which derive from the fusing of gametes, SEMs are formed from pluripotent stem cells (PSCs), including embryonic stem cells (ESCs) and induced pluripotent stem cells (iPSCs). This development has made it possible to investigate early embryogenesis using fresh approaches free from the ethical restrictions relevant to human embryos [3]. Techniques for generating SEMs include self-organizing stem cell aggregation, blastoid development, gastruloid growth, and trophoblast integration [4]. These techniques allow the study of developmental processes in vitro, providing an understanding of organogenesis, cellular differentiation, and early lineage specification (Fig. 1).

Natural and synthetic embryos differ mainly in their developmental potential and structural completeness. While SEMs may copy many features of early development, their inadequate extraembryonic support systems prevent them from becoming live entities [5]. Notwithstanding these constraints, SEMs provide valuable platforms for congenital disease modeling, pharmacogenomic applications, and tissue engineering. Using patient-derived iPSCs, researchers can design customized models for studying genetic defects, metabolic problems, and neurodegenerative diseases [6].

Integration of multi-omics approaches—including single-cell transcriptomics, epigenetics, and proteomics—has improved the application of SEMs. Developments in artificial intelligence (AI) and computer modeling have made predictive analyses of developmental paths easier and improved experimental conditions possible for more repeatability [7]. Furthermore, by permitting exact changes, CRISp-Cas9 gene editing helps research gene function and disease etiology [8]. But the emergence of SEMs calls serious ethical and legal questions. These models still have great moral debate since they mimic early-stage embryos but lack complete developmental capacity [9]. Rules must change to support SEM research while upholding moral scientific norms. Social issues with embryo research call for ethical control and open communication. Despite these obstacles, SEMs show great promise to improve biomedical research. Compared to conventional stem cell treatments, which sometimes suffer from immunogenicity and tumorigenicity, stem cell therapies produced from patient-specific induced pluripotent stem cells (iPSCs) may provide improved safety and compatibility [10]. Though preclinical research is still in its early stages, the ongoing study seeks to improve synthetic embryo technology for possible medicinal uses. Future developments in organoid complexity, developmental faithfulness, and global ethical standards for SEM research could be in store.

This work intends to thoroughly review current developments in synthetic embryo models, stressing the approaches applied in their production, their uses in scientific study, and the ethical and legal issues related to their use. Using a critical comparison between conventional stem cell research and SEM research, this review examines the function of iPSCs in generating synthetic embryos. It assesses whether this technology might alleviate worries about immunogenicity and tumorigenicity related to standard stem cell therapies. By SEMs, we investigate using artificial intelligence with new omics technologies, including transcriptomics, single-cell genomics, and epigenetics, to progress pharmacogenomics, regenerative medicine, and personalized medicine. Because SEMs have a transforming power in treating genetic diseases, neurological illnesses, and metabolic problems, this review is a crucial tool for academics looking for the most recent knowledge on the fast-developing area. Examining current preclinical and clinical research, we describe the future course of SEM technology and assess its possible benefits and drawbacks.

## Stem Cells: from Synthetic Embryos to Blastoids and Beyond

Rising as a transforming tool in developmental biology, stem-cell-based embryo models (SCBEMs) let scientists mimic early mammalian embryogenesis in vitro. Whether induced pluripotent stem cells (iPSCs) or embryonic stem cells (ESCs), pluripotent stem cells—guided to self-organize into structures that closely resemble those present in normal embryos—are used to build these models. This self-organization is directed by the exact regulation of biochemical and biophysical cues guiding the differentiation of stem cells into particular embryonic lineages. By changing signaling pathways and the extracellular matrix environment, it is possible to drive stem cells to create ordered structures that replicate the temporal and spatial patterns seen in normal embryonic development. Providing a dynamic environment for exploring the molecular basis of early development, these SCBEMs have effectively recreated critical developmental milestones, including organogenesis, germ layer development, and symmetry breaking. The use of SCBEMs has significant consequences for fundamental research and translational uses. Historically limited by the availability of human embryos, these models offer a reproducible and regulated system that provides a more complete study of early developmental processes. SCBEM research can improve assisted reproductive technologies, clarify the causes of developmental abnormalities, and guide preventative policies for congenital malformations. Furthermore, the possibility of imitating human development in vitro opens doors for pharmacological testing and toxicity evaluations in a setting quite similar to human physiology. Apart from improving our knowledge of human embryogenesis, as research develops, SCBEMs are expected to support creative therapeutic approaches, including regenerative medicine methods and the development of individualized medical care [11].

Thanks to recent developments in stem cells and developmental biology, there are now unheard-of possibilities to study embryogenesis using in vitro models that faithfully reproduce early mammalian development. These models were produced with thorough knowledge of stem cell biology and the ideas guiding embryonic development. Their origin is human and mouse stem cells. By following historical history in this discipline, researchers have created in vitro systems replicating many phases of embryonic development, from pre-implantation to early organogenesis. These models provide a framework for looking at developmental abnormalities and congenital diseases and help us to understand normal developmental processes. The fabrication of synthetic blastoids and embryos has offered new opportunities for translational science research. These SCBEMs can identify the fundamental causes of developmental abnormalities and guide preventative actions by providing a controlled environment for analyzing the molecular pathways under early development. Furthermore, by clarifying tissue differentiation and organogenesis, SCBEMs can drive assisted reproduction technologies and regenerate medicine. As this field develops, these creative models are expected to improve our knowledge of human development and support new treatment approaches and customized medical interventions [8].

With an eye toward the first two weeks following conception, Shahbazi and Pasque look at the early phases of human development in their 2024 review. They review the creation of stem cell lines that replicate early embryonic stages and look at the several cell lines that arise now. This paper clarifies human embryogenesis and the possible application of stem cell-based models in reproductive medicine and developmental biology. The writers also stress the progress in producing human embryo models from stem cells that copy several traits of early human development. Although they provide insightful analysis of the processes controlling embryogenesis, these models have great potential for exploring developmental anomalies and evaluating possible therapy approaches. This review connects in vitro modeling with in vivo embryology to underline the importance of stem cell-based technologies in improving our knowledge of human development [12].

Recent studies have clarified how synthetic embryos produced from stem cells self-assemble, stressing the critical functions of cadherin-mediated cell adhesion and cortical tension. Within mammalian development, the epiblast, trophectoderm, and primitive endoderm lineages match embryonic stem (ES), trophoblast stem (TS), and extraembryonic endoderm (XEN) cells, respectively. These stem cells can self-organize into structures that look like post-implantation embryos. The expression of cadherins, a class of calcium-dependent cell adhesion molecules, determines these cells’ spatial arrangement and varies with lineage. XEN cells show a unique cadherin profile that allows them to orient themselves under ES cells, therefore matching the arrangement of the primitive endoderm concerning the epiblast in genuine embryos. TS cells have cadherin expression that guides their orientation over ES cells, much as the trophectoderm naturally positions itself over the epiblast. Differential cadherin expression drives exact cell sorting that defines the basic architecture of the growing embryo. Together with cadherin-mediated adhesion, the preservation of tissue architecture throughout synthetic embryogenesis depends on the cortical tensional force produced by the actomyosin cytoskeleton under the cell membrane. Cortical stress improves the organization of structured elements after the first cell sorting by affecting mechanical characteristics and cell shape. Using experimental manipulation of cortical tension and cadherin expression, researchers have shown that they may improve the formation efficiency of well-organized synthetic embryos. These findings enhance our knowledge of the basic rules controlling embryonic development and help advance stem cell-based models, which have superb possibilities for studying developmental processes and modeling diseases [13].

Researchers co-cultivated two extraembryonic-like cells modified to overexpress particular transcription factors with wild-type human embryonic stem cells in a recent effort to produce a human embryoid model that nearly resembles the post-implantation human embryo. This creative method has created a three-dimensional structure replicating essential aspects of early human development by combining extraembryonic and embryonic components. Due to ethical and technical restrictions, the model offers a unique forum for tracking and evaluating the complex morphogenetic changes following post-embryo implantation. The study emphasizes the need for tissue-tissue contact in early development by showing that extraembryonic-like cells greatly influence the differentiation of the epiblast-like domain by changing the subpopulations within the hypoblast-like compartment. Basic and translational research significantly impacts this flexible and modular human embryoid model. The great frequency of pregnancies ending at this level allows scientists to investigate essential questions about human post-implantation development. Examining these mechanisms in vitro offers vital new perspectives on the causes of early pregnancy loss and congenital abnormalities, therefore helping to guide the development of preventative policies. This model provides a flexible framework for evaluating the impact of different drugs and environmental factors on early human development, improving toxicology and reproductive medicine. As research advances, the human embryoid model could become an increasingly important tool for broadening our knowledge of human embryogenesis and enhancing clinical outcomes related to early development [14].

In a landmark finding, scientists showed that led by an organizer, an artificial morphogen signaling center, clusters of mouse embryonic stem cells may create embryo-like structures, sometimes called “embryoids.” Specific morphogens emitted by this organizer direct stem cells’ spatial arrangement and differentiation, producing structures closely reflecting the first phases of mammalian embryonic development. The successful development of these embryoids indicates the ability of localized signaling centers to coordinate complicated tissue patterning and morphogenesis throughout embryogenesis. This progress clarifies early developmental mechanisms and provides a consistent in vitro model for examining embryonic development and related illnesses [15].

Researchers have made a noteworthy discovery by proving that mouse embryonic stem cells (ESCs) could be reprogrammed to produce embryo-like structures, sometimes known as embryoids, which grow from the rosette to the lumen stage. Inducing specific transcription factors helps to accomplish this reprogramming by effectively guiding embryonic stem cells to self-organize into structures roughly approximating early embryonic development. The studies prove that transcription factor-mediated reprogramming may direct embryonic stem cells to generate complex structures, offering a major in vitro model for studying early developmental events [16].

Thanks to the regeneration of their complete genomes, advances in genetic science allow the prediction of common disease risks in human preimplantation embryos. This procedure examines embryos and studies parent genomes to find inherited genetic variations. Scientists can evaluate embryo susceptibility to several common disorders by identifying uncommon mutations and computation of polygenic risk scores (PRS). This idea presents a complete approach for assessing disease risk before implantation, improving individualized treatment, and maybe directing reproductive decisions. Still, using whole-genome risk prediction in preimplantation embryos begs moral and scientific questions. Though it offers insightful analysis of possible health effects, studies are under progress to evaluate the validity and practicality of these forecasts. Experts warn that as the study is still young, the accuracy of these approximations for complex diseases is currently unknown. Ethical and social consequences have drawn criticism, including the possibility of eugenics and prejudice. As this discipline develops, the advantages of sickness risk prediction need careful evaluation concerning ethical criteria and scientific integrity [17]. The current usage of stem cells’ models in regenerative medicine including advantages and disadvantages are summarized in Table 1.

**Table 1:** Current usage of stem cell models in regenerative medicine

Stem Cell Type/Model	Current Applications	Advantages	Disadvantages
Embryonic Stem Cells (ESCs)	- Cardiomyocyte regeneration - Spinal cord injury repair - Diabetes treatment research	- Pluripotent: differentiate into all cell types - Stable proliferation	- Ethical concerns - Risk of teratoma formation - Immune rejection
Induced Pluripotent Stem Cells (iPSCs)	- Disease modeling (e.g., Parkinson’s, AD) - Drug screening - Personalized regenerative therapies	- Avoids ethical issues - Autologous compatibility - Pluripotency retained	- Genetic instability - Reprogramming inefficiency - Potential tumorigenicity
Mesenchymal Stem Cells (MSCs)	- Bone and cartilage repair - Treatment of inflammatory diseases - Cardiac regeneration	- Immunomodulatory - Easily isolated (e.g., from bone marrow, adipose tissue) - Low immunogenicity	- Limited differentiation potential - Senescence in culture - Variable efficacy
Neural Stem Cells (NSCs)	- Treatment for neurodegenerative diseases - Stroke recovery - Spinal cord injury repair	- Neurogenic potential - Potential for CNS repair	- Limited source availability - Low proliferation rate in vitro
Hematopoietic Stem Cells (HSCs)	- Bone marrow transplantation - Treatment for blood disorders (e.g., leukemia, thalassemia)	- Established clinical use - Robust engraftment	- Graft-versus-host disease risk - Limited to hematopoietic lineage
Very Small Embryonic-Like Stem Cells (VSELs)	- Regenerative potential across multiple tissues - Anti-aging and anti-cancer research	- Pluripotent-like - Present in adult tissues - Non-tumorigenic	- Controversial identity - Difficult to isolate and expand
Synthetic Embryo Models (SEMs)	- Modeling implantation - Studying early embryogenesis - Reproductive medicine	− 3D structure mimics natural development - Allows ethical exploration of early events	- Not fully equivalent to embryos - Limitations in modeling later development
Organoids from Stem Cells	- Brain, liver, kidney regeneration studies - Disease modeling - Drug response prediction	− 3D architecture - Patient-specific organoids for precision medicine	

## Methodologies in Synthetic Embryo Model Generation

Simulating artificial organs depends critically on the aggregates of pluripotent stem cells. Essential platforms for studying early developmental processes, embryoid bodies (EBs), and gastruloids copy particular features of germ layer differentiation. Though they lack spatial order, embryoid bodies (EBs), produced by the spontaneous aggregation of embryonic stem cells (ESCs) or induced pluripotent stem cells (iPSCs), show traits reminiscent of early embryonic development [18]. On the other hand, because of their improved organizational order and better axial patterning representation, gastropods help explore early morphogenetic events [19].

Researchers have introduced extraembryonic lineages and trophoblast into SEMs to improve developmental fidelity. Co-culture techniques include extraembryonic endodermal cells and trophoblast-like cells to enhance the fidelity of embryogenesis replication. These models help embryonic elements to be correctly organized and strengthen placental contacts. Including extraembryonic elements improves synthetic embryos’ physiological significance, bridging the gap between natural dives [20].

Two organoid-based models that have progressed in synthetic embryology are amnioids and blastoids. Like blastocysts, blastoids are stem cell-derived entities that offer an in vitro model for examining early lineage differentiation and implantation [21]. In line with this, amniotic systems try to recreate whole embryonic structures—including extraembryonic compartments—to clarify developmental mechanisms following implantation [22]. These models have a great capacity to understand difficulties with placental dysfunction and implantation.

Single-cell technologies and gene editing have transformed the research on synthetic embryos. Because of CRISp-Cas9’s capabilities, researchers can examine gene expression during early development [23]. Single-cell transcriptomics offers high-resolution views of gene expression patterns, helping one grasp cellular differentiation and lineage commitment [24]. These technologies, taken together, accelerate developments in embryology and regenerative medicine.

Lau et al. (2023) outlines a thorough approach for in vitro generation of whole mouse embryo models using both embryonic and induced pluripotent stem cells. This approach helps build intricate, three-dimensional models that closely resemble actual mouse embryos, improving the study of early developmental processes. The method defines the conditions and actions needed to direct stem cells in self-organization and differentiation, producing models that reflect essential aspects of embryogenesis. This development gives scientists a valuable tool to look at the processes behind diseases and development, therefore providing information that can influence regenerative medicine and treatment approaches [25].

Thanks to advances in stem cell biology, in vitro models that faithfully replicate human peri-implantation development can be created, offering a critical new understanding of early embryogenesis. Researchers have produced three-dimensional human embryos and embryo-like constructions replicating essential aspects of the post-implantation phases. These models help to understand cellular composition, gene expression, and lineage differentiation over early development. By studying these structures, researchers can improve their understanding of the processes behind organogenesis, tissue patterning, and implantation. Understanding developmental problems and enhancing reproductive health both depend on these systems. Developing these in vitro models will significantly affect fundamental and functional biomedical research. They provide a forum for looking at human-specific developmental processes that are difficult to observe in vivo because of ethical and technological restrictions. Using these models, one can explore the effects of pharmacological drugs, environmental elements, and genetic variations on early development, thereby facilitating the discovery of possible therapeutic targets and the progress of individualized medicine practices. While expanding our knowledge of human development as the research advances, these embryo-like assemblies can improve treatment results for developmental diseases and reproductive health [14].

A new study identified a vital network of nuclear receptors and SINE B1 components controlling enhanced pluripotency in blastoids and blastocysts spanning several species. Early embryonic development depends on this natural program, which also influences the pluripotent state of stem cells during the construction of these structures. Understanding this network provides important new angles on the molecular mechanisms controlling early development and pluripotency. Recent studies have revealed a critical network controlling increased pluripotency in blastoids and blastocysts of several species. Comprising nuclear receptors and SINE B1 components, this network, the intrinsic program required for early embryonic development, shapes stem cells’ pluripotent state while building these structures. Learning about this network helps one understand the molecular processes controlling early development and pluripotency [26]. Recent studies efficiently generated monkey blastoid capsules from aged somatic cells, addressing monkey blastoid cavies’ generally low development efficiency, usually sub 30%. This result could significantly improve our knowledge of early primate development and progress in regenerative medicine [27].

### Bioengineering Techniques to Improve Synthetic Embryo Models (SEMs) Progress

Recent developments in bioengineering have significantly enhanced the building of SEMs, hence enabling a more exact reproduction of early embryonic structure, tissue organization, and functional dynamics. These developments tackle major gaps in synthetic biology, reproductive medicine, and developmental biology.

1. Organ-on-a-chip systems and microfluidic

Microfluidic technologies have been absolutely vital in controlling the spatial and temporal microenvironment of stem cells by mimicking in vivo gradients and allowing dynamic culture conditions. Accurate nutrition flow, morphogen distribution, and mechanical stimulation provided by these platforms are vital for guiding lineage specification and symmetry breaking during early development [28, 29].

2. Three-Dimensional Bioprinting and Biomaterial Scaffolds

Hydrogel-based biomaterials—such as Matrigel, PEG, and fibrin—provide a supportive extracellular matrix (ECM)-analogous environment that helps cells self-organize into embryonic-like structures. Recent developments in 3D bioprinting have enabled the spatially controlled deposition of matrix components and stem cells, hence producing multilayered structures resembling embryos with improved morphological fidelity and lineage patterning [20, 30].

3. Optogenetics and Synthetic Morphogen Gradients

The integration of synthetic biology technologies, including inducible genetic circuits and optogenetics, has enabled precise regulation of gene expression and morphogen signaling pathways (e.g., WNT, BMP, NODAL). These methods increase the uniformity of SEM generation across tests and enable coordinated developmental programs [31, 32].

4. Combining Transcriptomics with CRISPR-Enhanced Lineage Tracing

SEM systems have included CRISPR barcoding and advanced single-cell transcriptomics to monitor lineage commitment and evaluate cell fate dynamics. This allows real-time mapping of embryoid growth and attachment to natural embryo progression, hence providing feedback to improve model design progressively [5, 33].

5. AI-Enhanced Morphogenesis and Automation

Machine learning techniques and artificial intelligence (AI) are used to predict and track embryoid morphology and fate decisions. Predictive modeling and automated image analysis together enhance quality control and allow broad SEM screening across several bioengineering settings [34, 35]. These several bioengineering successes are pushing SEMs toward physiologically suitable embryo analogs. The continuous synthesis of synthetic biology, microfluidics, and materials science provides possibilities for improving SEMs to address congenital illness models, implantation failure, and infertility.

## Potential Applications of Synthetic Embryo Models

Synthetic embryo models (SEMs) have extensively driven stem cell and developmental biology advances. Whether derived from embryonic stem cells (ESCs) or induced pluripotent stem cells (iPSCs), these in vitro models reflect critical phases of early development and provide hitherto unheard-of research and therapeutic possibilities. By overcoming the ethical and practical constraints connected with natural embryos, SEMs have offered fresh approaches to understanding human development, developing regenerative medicine, and solving worldwide disease modeling and infertility difficulties. One of SEMs most important uses is in clarifying the intricate dynamics of early human development. Ethical restrictions on the use of human embryos in research and their inaccessibility have rendered the post-implantation phase of embryogenesis, also known as a “black box,” little understood. Gastruloids and blastoids, among other SEMs, offer a controlled and morally acceptable framework for looking at this critical phase. Today, scientists can look at the molecular and cellular processes behind gastrulation, neurulation, and lineage specification [36, 37]. Recent developments allow human SEMs to replicate the spatial architecture of embryonic and extraembryonic tissues precisely. These findings are essential for embryonic biology and can advance assisted reproductive technologies (ART) and lower early pregnancy loss, common in the first phases of development [37].

In regenerative medicine, SEMs show transforming power. By mimicking early embryogenesis, scientists can improve their understanding of tissue formation and organogenesis, thereby fostering genetically similar tissues and organs for transplantation. Human heart development has been investigated using Scanning Electron Microscopes (SEMs), offering an understanding of possible tissue regeneration techniques and congenital cardiac abnormalities [38]. Furthermore, in vitro gametogenesis (IVG) can transform reproductive therapy by producing functional gametes from induced pluripotent stem cells (iPSCs). Although IVG has been effectively shown in mice, SEMs offer a way to overcome the difficulties of reproducing these findings in humans, including the ethical questions of human embryo research and the scarcity of human gonadal somatic cells [37]. encouraging postmenopausal motherhood and the treatment of infertility can help to solve a significant health concern influencing 17.5% of the adult population worldwide [37]. SEMs are promising for pharmaceutical development and illness modeling. By copying early embryonic stages, these models allow one to investigate the origins of genetic diseases and developmental abnormalities. Investigating the source of primordial germ cells using scanning electron microscopes (SEMs) has provided an understanding of issues including germ cell cancers and infertility [36]. Creating patient-specific SEMs from iPSCs helps researchers examine the safety and efficacy of medications using models that closely reflect the patient’s genetic composition, therefore enabling tailored medicine practices. For those with rare or complicated diseases, this could improve results and significantly save the time and expenses of clinical studies [8].

Even if SEMs have certain advantages, their use begs serious ethical questions. Conventional ideas of embryogenesis are questioned, forcing a review of current ethical guidelines since the creation of embryo-like beings without eggs or sperm challenges them. Although SEMs cannot develop into live entities, their striking similarity to actual embryos has sparked discussions about their ethical consequences and application [39]. Establishing explicit rules and control systems that combine ethical integrity with scientific advancement will help to guarantee that SEM research is carried out clearly and ethically.

In their 2022 work, Dong et al. performed a thorough genome-wide CRISp-Cas9 knockdown screen to find growth-limiting and vital genes in human trophoblast stem cells (hTSCs). This methodical approach revealed essential genes required for ESC self-renewal and proliferation, offering a critical new understanding of the molecular processes controlling trophoblast development. The results might guide therapy plans for disorders related to trophoblasts and significantly improve our knowledge of placental biology [40].

**Fig. 2:**
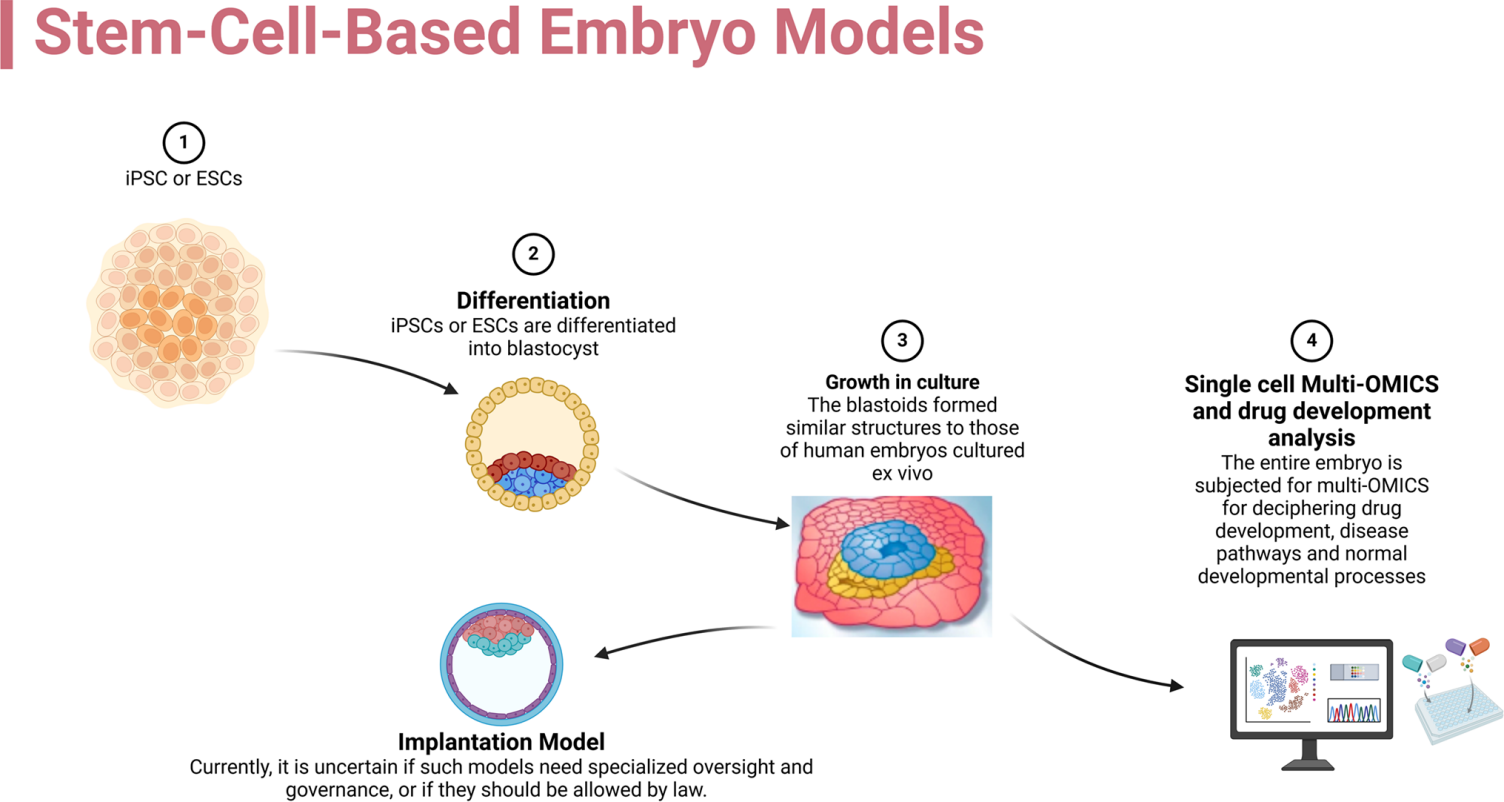
Stem-cell-based embryo models, or SCBEMs and potential downstream analysis: SCBEMs have emerged as a revolutionary technique in developmental biology. These models are created by guiding pluripotent stem cells, either embryonic stem cells (ESCs) or induced pluripotent stem cells (iPSCs), to self-organize into structures that closely mirror those of genuine embryos. This self-organization is orchestrated by precise modulation of biochemical and biophysical cues that direct the differentiation of stem cells into discrete embryonic lineages. After SCBMS form, they may undergo several downstream analyses, including drug development, single-cell multi-omics, and the interpretation of typical developmental events. It is unclear whether these models require professional control and governance or whether the law should let them

Researchers recently developed a molecular clock method using cell state and lineage data to record clonality in vivo and the timing of biological processes. This approach used a recently created multipurpose single-cell CRISPR platform, NSC-seq, to precisely monitor tissue-specific cell expansion during mouse embryonic development and identify new intestinal epithelial progenitor states based on their genetic histories. The study investigating human precancers and mouse adenomas discovered that 15–30% of colonic precancers had several regular founders, suggesting poly-ancestral origin. Enhancing current single-cell studies and providing the basis for in vivo multimodal recording, this multimodal technique helps to monitor lineage and temporal events during development and cancer. The study results significantly impact our knowledge of the development and etiology of precancerous lesions. The study clarifies the intricacy of early carcinogenesis by proving the poly ancestral source of colonic precancers and implying that numerous normal cells rather than a single progenitor may cause these disorders. This realization might improve the subsequent early detection of colorectal cancer and intervention plans. By allowing researchers to track the lineage and timing of cellular changes in vivo, the NSC-seq method provides a potent tool for the temporal documenting of cellular events, improving our knowledge of developmental biology and disease progression [41]. Figure 2 shows the downstream study and expected results of embryo models created from stem cells. Following the SCBMS form, they could undergo many downstream analyses covering drug development, single-cell multi-omics, and standard developmental event interpretation. The need for professional supervision and governance of these models is still debatable, as is the issue of whether legal systems should let them flourish.

## Applications of Synthetic Embryo Models in Disease Research and Therapy

### Disease Modeling

Synthetic embryo models (SEMs) in biomedical research have emerged as a revolutionary instrument, offering unprecedented insights into the cellular and molecular mechanisms behind complex diseases such as cancer, diabetes, cardiovascular conditions, and neurodegenerative disorders. By modeling early embryonic phases, SEMs offer a framework for examining disease causation, identifying potential treatment targets, and developing diagnostic techniques. With modern research and important molecular routes found by their use, this part investigates the possible uses of SEMs in several fields.

One of the defining traits of neurodegenerative illnesses, including Parkinson’s and Alzheimer’s, is the slow decrease of neuronal activity. To grasp the pathophysiology of these diseases, researchers have investigated the early phases of brain development, including neural tube formation—using scanning electron microscopes (SEMs). SEMs have been employed to mimic the development of the anterior brain, an area often affected by neurodegenerative illnesses, by replicating the signaling exchanges between embryonic and extraembryonic tissues. As a result, the Wnt/β-catenin pathway has been found among other vital genes and signaling pathways linked to neurodegeneration and neural tube abnormalities. Therapeutic strategies are interesting since present studies assess medications that might affect these pathways using SEMs [42]. Using brain organoids as models for human brain development and illness was explored. The advantages and disadvantages of these models and the stress of how faithfully they can replicate the complexity of the human brain in vitro were discussed. The value of brain organoids in investigating many facets of neurodevelopment—including cortical patterning, neural circuit building, and neuronal differentiation. The study examines how organoids might be used to replicate neurological diseases such as Parkinson’s and Alzheimer’s, therefore offering an understanding of their fundamental causes and possible treatments. By combining modern research results, the study stresses the importance of brain organoids in improving our knowledge of human brain biology and their possibilities in tailored treatment [43].

A significant cause of death globally is cardiovascular diseases (CVDs). Using scanning electron microscopes (SEMs) has dramatically enhanced research on early heart development, including establishing the beating heart tube and the differentiation of cardiac progenitor cells. SEMs have been used by researchers to replicate congenital heart disorders and clarify the molecular mechanisms engaged, including the function of the Notch signaling cascade in cardiac cell fate determination [36]. SEMs have also facilitated the development of patient-specific drug screening models, which can help identify compounds capable of regenerating or healing heart tissue. These advances could change how CVDs are treated by providing tailored therapeutic options.

Recent work indicated that clonogenic endothelial-macrophage (EndoMac) progenitor cells comprised the adventitia of the animal aorta at both embryonic and postnatal periods. Originating from an early embryonic CX₃CR1⁺ and CSF1R⁺, these bipotent progenitors are independent of Flt3-mediated bone marrow hematopoiesis. Their proliferative and vasculogenic characteristics help perfused blood vessels to develop and support adventitious neovascularization when they enter ischemic tissue. Furthermore, the study shows how angiotensin II controls the increase of their clonogenic and differentiating characteristics, enabling their proliferative expansion in vivo. According to the research, endoMac progenitors produced from embryos help postnatal development of endothelial cells and macrophages, which then participate in local vasculogenic reactions inside the aorta wall. This discovery will substantially change our knowledge of vascular biology and possible therapeutic approaches. By clarifying the function of EndoMac progenitors in vascular growth and repair, the research investigates paths for regenerative medicine treatments targeted at vascular diseases. The discovery of these progenitors clarifies the biological sources of aortic endothelial and macrophage populations, guiding the following studies on tissue engineering and stem cells [44].

Through a rigorous study of many cell types within the heart, single-cell transcriptomics has transformed our knowledge of cardiac diseases by exposing cellular diversity, identifying particular transcriptional signatures linked with heart failure and aging, and underlining the emergence of disease-related cellular states. Using gene expression profiles of individual cells, researchers have discovered new cellular populations and their dynamics in the formation of the human heart. Combining suspension-based and innovative in situ techniques, this thorough investigation offers insights into the cellular environment of the heart. Thanks to single-cell RNA sequencing, a new understanding of disease etiology shows how the endothelium controls cardiomyopathies, including non-compaction. These results highlight how well single-cell transcriptomics might improve our heart biology and pathophysiology knowledge. Finally, SEMs and single-cell transcriptomics have produced a thorough map of the heart’s cellular makeup, enabling individualized treatment plans and providing a critical new understanding of the molecular mechanisms behind cardiac disorders [45].

Recent work using a genome-wide CRISPR screen in human induced pluripotent stem cells (hiPSCs) undergoing differentiation into CMs has found BRD4, a member of the bromodomain and extra terminal (BET) family, to be a fundamental regulator of cardiomyocyte (CM) differentiation. Interacting with BRD4, acetylated lysine residues on histones and non-histone proteins affect chromatin remodeling and gene expression. The modification of BRD4 activity greatly affected the efficiency and development of cardiomyocyte differentiation from human-iPSCs. These results show that BRD4 is essential for cardiac lineage commitment and might be the focus of treatments to improve heart regeneration [46].

Current studies using SEMs expand cardiac tissue engineering methods, human-specific models for neurological diseases, and functional pancreatic beta cells generated for diabetes treatment [36, 39]. Significant results include the development of patient-specific organoids for tailored therapy, disease-specific biomarkers, and high-throughput drug screening systems. Early diagnosis approaches have their roots in finding molecular markers linked with the early phases of Alzheimer’s disease, which was made possible by scanning electron microscopes (SEMs) [42].

Stem cell research has advanced the construction of in vitro models that faithfully reflect human peri-implantation development, offering a critical new understanding of early embryogenesis. Researchers’ three-dimensional human embryos and embryo-like constructions reflect essential aspects of the post-implantation phases. These models help to analyze cellular composition, gene expression, and lineage differentiation over early development. By studying these structures, researchers can better understand the processes controlling organogenesis, tissue patterning, and implantation. Understanding developmental disabilities and enhancing reproductive health depends on these systems. Developing these in vitro models will significantly affect fundamental and functional biomedical research. They provide a forum for looking at human-specific developmental processes that are difficult to observe in vivo because of ethical and technological restrictions. Using these models, one can explore the effects of pharmacological drugs, environmental elements, and genetic variations on early development, therefore supporting the discovery of possible therapeutic targets and the progress of individualized medicine practices. As science advances, these embryo-like assemblies can improve therapy outcomes for developmental diseases and reproductive health and deepen our knowledge of human development [47].

SEMs have clarified critical molecular processes behind the start of diseases. Understanding the function of epigenetic changes in neural development helps explain the causes of neurodegenerative illnesses [42]. The discovery of the interaction between mechanical and chemical signals in cardiac development has led to fresh approaches for treating congenital heart diseases. These results highlight how SEMs have transforming power in clarifying illnesses’ cellular and genetic causes. All told synthetic embryo models are a powerful tool for improving our understanding of complex diseases and developing new treatment strategies. By trying developmental biology with disease research, SEMs are helping to advance neurological diseases, cardiovascular diseases, diabetes, and cancer. As research develops, using SEMs in clinical practice shows excellent possibilities for improving patient outcomes and addressing significant challenges in modern medicine.

Artificial embryo models are a novel instrument with great use in disease research, regenerative medicine, and developmental biology. By revealing the first phases of human life, SEMs can transform our knowledge of embryogenesis, enhance reproductive treatments, and progress tailored medicine. Strong ethical frameworks should direct their application to handle the several moral dilemmas they offer. As this field develops, SEMs will undoubtedly be very important in determining the course of science and medicine since they provide hope for solving essential problems in human health. An excellent tool for improving our knowledge of complicated diseases and developing creative treatment plans is artificial embryo models. By tying developmental biology with illness research, SEMs help to advance diabetes, cancer, cardiovascular diseases, and neurodegenerative disorders. Applying SEMs in clinical practice can improve patient outcomes and solve essential issues in contemporary medicine as research advances.

### Regenerative Medicine

The ability of synthetic embryo models to faithfully reproduce early developmental events has important consequences going beyond disease modeling into regenerative medicine. These models provide a strong basis for the creation of creative therapeutic approaches by imitating important components of embryogenesis, therefore revealing major insights on cellular differentiation, tissue organization, and organogenesis. Production of patient-specific cells and tissues provides possible answers for organ transplantation, damage repair, and degenerative disorders. Incorporation of these models into regenerative medicine may help researchers improve tissue engineering and tailored treatment by means of more closely depicting human development.

The difficulties and possible usage of pluripotent stem cell-derived cardiomyocytes (PSC-CMs) in cardiac regeneration were investigated. Good differentiation techniques are crucial to producing mature, functioning cardiomyocytes that fit well with the host myocardium. They underline the need to develop plans to improve electrical connectivity to current cardiac tissue, engraftment, and cellular survival. The study discusses improving cell output to satisfy clinical needs and guarantees PSC-CM’s safety to stop arrhythmias or carcinogenesis. Notwithstanding these obstacles, scientists hope PSC-CMs will transform heart regeneration treatments. The study emphasizes the need to tackle these difficulties to maximize the therapeutic possibilities of PSC-CMs in cardiac repair properly. By enhancing stress differentiation techniques, improving cell survival and integration, and guaranteeing safety, researchers can forward the development of efficient regenerative treatments for heart illness. The study support more multidisciplinary cooperation to go beyond these challenges and advance the clinical implementation of PSC-CM-based treatments [48].

Stem cell models, particularly those utilizing human induced pluripotent stem cell-derived cardiomyocytes (iPSC-CMs), have demonstrated significant utility in investigating genetic arrhythmia. These models help scientists understand the underlying causes of different arrhythmic diseases by reproducing particular genetic abnormalities in vitro. Developing iPSCs from individuals with hereditary arrhythmias into cardiomyocytes allows researchers to evaluate disease features and examine the effects of possible therapeutic therapies. Using iPSC-CMs in disease modeling offers the possibility for high-throughput medication screening and patient-specific cellular response analysis. Still, challenges remain, including the exact reproduction of the complex tissue architecture of the heart and the attainment of uniform differentiation processes. Notwithstanding these challenges, continuous developments in gene editing and stem cell research could improve these models’ accuracy, expanding our knowledge of hereditary arrhythmias and helping to develop individualized treatment plans [49].

The genes are linked to structural heart anomalies, congenital cardiac diseases, and cardiomyopathy. Using sophisticated genomic approaches, the researchers identified genes linked to many diseases, offering critical new perspectives on their molecular etiology. This all-encompassing gene finding improves our knowledge of the genetic elements linked to cardiac disorders, guiding the following diagnostic and treatment plans [35].

SEM research has demonstrated benefits for diabetes, a metabolic condition marked by reduced insulin production or efficacy. The formation of pancreatic beta cells, which are accountable for insulin secretion, has been investigated using scanning electron microscopy (SEM). Researchers have identified essential transcription factors, including PDX1 and NKX6, that regulate beta cell development by simulating the differentiation of pluripotent stem cells into pancreatic lineages. This understanding has been useful for generating functional beta cells in vitro, potentially advancing treatment for type 1 diabetes through cell replacement therapy. Ongoing research concentrates on optimizing these procedures to guarantee the security and efficacy of transplanted cells [37].

SEMs have revolutionized cancer research by clarifying the function of embryonic signaling networks in carcinogenesis. An important mechanism in cancer metastases, the epithelial-to-mesenchymal transition (EMT), has been replicated with SEMs. Studies show that cancer cells reactivate active pathways like TGF-β and Hedgehog during early embryogenesis to drive invasion and metastases. These results have led to the formulation of targeted treatments meant to block these pathways, improving the prognosis for patients with aggressive malignancies [42].

Stem cell mechanics affect the development of epithelial tissues during cancer and pregnancy. The mechanical forces produced by stem cells influencing tissue form and function are under consideration, stressing their part in normal development and cancer. The need to know these mechanical interactions to improve cancer treatments and regenerative medicine is investigated. Using mechanical stress, including those between cells and between cells and the extracellular matrix, the authors probe the molecular pathways stem cells influence tissue patterning and homeostasis. They investigate how the deregulation of these mechanical processes can lead to diseases, including cancer, in which changed mechanical properties enable tumor development and spread. The study thoroughly explains how stem cell mechanics affect tissue dynamics by combining ideas from developmental biology and cancer research. Knowing stem cell behavior’s mechanical aspects could help develop fresh cancer and tissue engineering treatments. Targeting stem cell mechanical characteristics and their surroundings might help to promote tissue regeneration and stop tumor development. The study emphasizes the need for more research on the mechanical aspects of stem cell biology to develop efficient treatments for different diseases [50].

Synthetic embryo models’ fast-expanding field offers unmatched opportunities for studying human development, simulating illness progression, and looking at creative therapy techniques. These models provide a unique framework for investigating genetic diseases, congenital abnormalities, and the effect of environmental influences on early development by aggregating key components of embryogenesis. Moreover, they enable the evaluation of drugs and gene-editing technologies inside a physiologically relevant framework, therefore providing possibilities for customized therapies and regenerative medicine. Still, as research advances it also generates important ethical and legal questions. Synthetic embryo models challenge existing rules on human embryo research and demand a careful examination of scientific, ethical, and legal consequences to ensure responsible progress.

### Synthetic Embryo Models (SEMs) in Studying Implantation Failure and Pregnancy-Related Pathogenesis

Continued significant challenges to successful reproduction are caused by implantation failure and pregnancy-related problems such recurrent miscarriage, hypertension, and placental insufficiency. Rising as efficient tools for examining early embryonic and extraembryonic interactions vital for implantation, synthetic embryo models (SEMs) made from pluripotent stem cells (PSCs) devoid of fertilization.

SEMs can mimic fundamental peri-implantation events, ranging from the growth of epiblast, trophectoderm, and primitive endoderm-like compartments. Various configurations let scientists mimic the interplay of several lineages during the implantation phase and their interaction with maternal endometrial cells. Typically unsuitable or impractical in human embryos, SEMs offer high-resolution live imaging and genetic manipulation [4].

Many studies have demonstrated that SEMs precisely mimic normal embryos’ transcriptome and morphogenetic traits during the implantation phases. By correctly mimicking the start of early pregnancy, Fu et al. (2021) showed that mouse stem cell-derived embryo models produce implantation-competent structures able to cause decidualization both in vitro and in vivo [11]. Kagawa et al. (2022) successfully created human blastoids mimicking early post-implantation events, hence shedding light on the failures of trophoblast invasion and syncytiotrophoblast development, which are common in early pregnancy concerns [5].

Moreover, bioengineered endometrial platforms combined with SEMs offer a dynamic paradigm for exploring problems at the maternal-fetal interface. Aiding in the discovery of new therapeutic targets, these hybrid systems have shown how changes in gene expression or signaling pathways—such as WNT, BMP, and integrin signaling—affect implantation and early development [51].

Moreover, Liu et al. (2021) provide a system for generating human induced trophoblast stem cells (iTSCs), which might be included into SEMs to more exactly mimic placental development and examine its pathology [52].

SEMs are anticipated to revolutionize reproductive medicine by imitating implantation problems, clarifying molecular abnormalities, and assessing new treatments since they can mimic early human development within an ethically acceptable framework.

### Advantages of Synthetic Embryo Models (SEMs) Compared to Organoid Models

Though organoids have changed developmental biology and disease modeling by enabling the in vitro reproduction of organ-specific structures, their ability to mimic whole-organism developmental processes remains limited. There are several benefits of SEMs that clearly make them a better platform for studying implantation-related disorders and early development (Table 2).

**Table 2:** Comparison between synthetic embryo models (SEMs) and normal fertilized embryos

Feature	Synthetic Embryo Model (SEM)	Normal Fertilized Embryo
Origin	Assembled from pluripotent stem cells (e.g., ESCs, TSCs, XEN cells)	Formed via natural fertilization of oocyte by sperm
Genetic Composition	Often genetically identical (clonal ESC lines) or modified	Genetic recombination of maternal and paternal genomes
Totipotency	Lacks true totipotency; relies on pre-differentiated stem cell types	Zygote is truly totipotent and capable of forming all embryonic and extraembryonic tissues
Developmental Fidelity	Can mimic early stages (blastocyst formation, gastrulation, axis formation)	Complete developmental potential (embryo to fetus to full organism)
Extraembryonic Tissues	Reconstructed using XEN and TSCs to model yolk sac and placenta analogs	Develops naturally from trophectoderm and primitive endoderm
Implantation Potential	Limited implantation; can initiate decidual reaction in mice but does not develop into fetus	Fully implantable and capable of normal embryogenesis
Use in Research	Ideal for studying peri-implantation, lineage specification, and developmental diseases	Used in restricted settings; ethical and legal barriers limit availability
Ethical Status	Considered less ethically contentious (not derived from fertilization)	Subject to strict ethical guidelines and regulations
Availability	Can be mass-produced in vitro from stem cell lines	Limited availability; requires fertilization and consent
Applications	Modeling embryogenesis, implantation disorders, developmental biology, and regenerative medicine	Natural reproduction, IVF, and comprehensive embryological studies

1. Holistic Recapitulation of Embryogenesis

Organoids are often developed from a unique germ layer or lineage (e.g., brain, intestine, or hepatic), concentrating on producing discrete organ-like structures. Comprising several stem cell types—mostly embryonic stem cells (ESCs), trophoblast stem cells (TSCs), and extraembryonic endoderm (XEN) stem cells—SEMs enable the self-organization of embryonic and extraembryonic compartments, so emulating the whole conceptus comprising the epiblast, amnion, yolk sac, and trophoblast lineages [4, 51].

2. Modeling Embryo–Maternal Interface and Implantation

Organoids cannot mimic the implantation process or trophoblast invasion, both of which are crucial for understanding infertility and pregnancy-related disorders as preeclampsia. On the other hand, SEMs show blastocyst-like structures that can embed into uterine tissues, causing decidual reactions and mimicking peri-implantation developmental stages [5, 51]. This makes SEMs a necessary paradigm for studying interactions between mother and child.

3. Temporal and Spatial Patterning Fidelity

Encompassing symmetry breaking and germ layer specification, SEMs show sequential and spatial expression of developmental genes (e.g., OCT4, GATA6, CDX2) that closely match real embryonic development. Organoids often show variation in morphogen gradients and patterning, which leads to various and sometimes non-physiological differentiation outcomes [42, 53].

4. Systems-Level Integration and Axis Formation

Unlike organoids, which usually lack anterior-posterior (A-P) or dorsal-ventral (D-V) axes, SEMs can define embryonic axes and simulate gastrulation processes, hence enabling a more complete investigation of body plan development [54]. SEMs are very good at clarifying chemical signals connected to axis formation and symmetry breakage.

5. Ethical Advantages Over Natural Embryos

SEMs, produced from pluripotent and extraembryonic stem cells, provide an ethical substitute for human embryonic research by removing the need for fertilized eggs, thereby eliminating the need for fertilized eggs. For studies constrained by legal and ethical issues, SEMs are more beneficial since they not only avoid ethical limits but also offer a more complete view of early embryonic life [9].

### Primordial Germ Cells and Very Small Embryonic-Like Stem Cells (VSELs) in Synthetic Embryo Models

The embryonic precursors of gametes, primordial germ cells (PGCs), need exact specification and migration for developmental competency and intergenerational continuity. Ideally, SEMs that efficiently mimic early embryonic processes should include the development of primordial germ cells (PGCs) to be functionally equivalent to natural embryos.

Recent research indicates that SEMs—especially those derived from ESCs in combination with extraembryonic stem cell types (such as trophoblast stem cells and extraembryonic endoderm stem cells)—can mimic early lineage specification events, including the generation of PGC-like cells (PGCLCs). PGCLCs in mouse SEMs have been reported to grow under specific in vitro conditions mimicking gastrulation and epiblast signaling microenvironments. These SEMs can show early migratory activity towards extraembryonic locations, replicating their in vivo equivalents, and express typical PGC markers like as Blimp1, Prdm14, and Stella.

In SEMs, the presence and distribution of these cells in all developing tissues is varied and context dependent. Unlike natural embryogenesis, where PGCs grow in a controlled temporal and spatial way, modern stem cell models can call for external induction signals or improved bioengineering settings to consistently promote PGC development [8, 42, 55, 56].

This subject is strongly related to the developing idea of very small embryonic-like stem cells (VSELs). VSELs are a putative population of pluripotent stem cells identified in adult tissues with qualities similar to early embryonic cells, including the ability to trigger tissue regeneration and cancer. VSELs are thought to represent a latent pool of cells from early embryonic phases comprising primordial germ cells (PGCs). Thetchinamoorthy et al. (2025) claim that because of their embryonic-like flexibility and persistence in adult organs, VSELs support tissue homeostasis and show promise in regenerative medicine applications [57]. Through their participation in cellular turnover and repair processes, Bhartiya et al. (2023) propose that VSELs might be crucial in tackling tissue damage, cancer progression, and aging [58].

VSELs and PGCs’ absence or inadequate representation in SEMs could compromise their developmental integrity. This emphasizes a vital area for future improvement in SEM research, especially the optimization of inductive cues, geographical compartmentalization, and niche engineering to support the formation and proper integration of PGCs and VSEL-like populations. Combining these elements could increase the translational potential of SEMs and provide fresh perspectives on disease models and early germline development.

## Ethical and Regulatory Considerations

Synthetic embryo models (SEMs) represent a revolutionary discovery in developmental biology and stem cell research, offering hitherto unheard-of possibilities to investigate human embryogenesis and tackle critical medical concerns. Still, their use creates serious ethical and legal questions, especially about their legal and moral status, experimental guidelines, possible reproductive uses, and society’s consequences. This essay looks at these issues in great detail, stressing the importance of strong systems to balance moral responsibilities with scientific development.

### Moral and Legal Status of Synthetic Embryo Models

SEM ethical evaluation depends on their moral and legal position. Unlike naturally occurring embryos produced by fertilization, SEMs are produced from pluripotent stem cells and cannot mature into live animals. This difference challenges accepted conceptions of embryogenesis and personality since SEMs blur the line separating biological models from things deserving moral attention. For instance, although SEMs can reproduce the first embryonic phases, such as gastrulation and organogenesis, most governments classify them as not embryos based on current legal rules [59].

As SEMs become more complex, their similarity to real-life embryos begs the question of when they might have moral status. Some contend stem cell models should receive safeguards similar to those given to normal embryos if they show features like a primitive streak or the possibility of continued development [60].

Further complicating this argument is the disagreement about the ethical relevance of early embryogenesis. Some traditions claim that embryos have full moral status from conception, while others maintain that moral status changes with time [61].

The current rules for SEM studies have been molded by the 14-day guideline, which caps research on human embryos at the first two weeks of development. Given the establishment of the primordial stripe, a key developmental milestone, this control was developed to balance ethical concerns with scientific investigation. Conversely, SEMs challenge this paradigm since their non-embryonic character allows them to be grown for more than 14 days without breaching any rules [62].

Recently changing its guidelines, the International Society for Stem Cell Research (ISSCR) advises that SEM research be assessed case-by-case and banned should it show a risk of ethical transgressions [63].

This method underlines ethical criteria and the need for flexibility in SEM research guidelines. The absence of clear global standards generates uncertainty and emphasizes the need for well-defined rules that consider SEMs’ special qualities [61].

### Potential for Reproductive Applications

One of SEM research’s most controversial parts is its possible reproductive applications. Although SEMs cannot develop live entities now, future technological developments could allow their application in assisted reproduction. Using SEMs, for instance, could help to replicate early pregnancy or develop functioning gametes, therefore providing creative solutions for reproductive health and infertility [64].

Still, great ethical questions surround the use of SEMs for reproduction. The development of artificial embryos for reproduction could commercialize human life and worsen already existing disparities in access to reproductive technologies [61]. Furthermore, even if it is still hypothetical, the possibility that SEMs could become living things calls for a thorough evaluation of the ethical consequences of such uses [60].

SEMs provide a fresh understanding of early embryonic development and help in infertility treatments, therefore transforming reproductive applications. Providing creative reproductive alternatives, researchers suggest simulating early pregnancy phases using SEMs, investigating implantation processes, and maybe developing functional gametes [65]. By allowing researchers to explore embryo viability, assess fertility therapies, and develop solutions for people dealing with infertility concerns, these models might help to enhance reproductive health [66].

Though its possible value, the use of SEMs in reproductive medicine creates serious ethical conundrums. Particularly in cases when synthetic embryos closely resemble human embryos in their embryonic stage, the ethical situation of these creations raises serious questions [9]. Some argue that the development of synthetic embryos hides the ethical differences between real human life and laboratory-generated entities, therefore undermining the established legal and moral systems meant to protect human embryos [67]. This forces important questions about the beginning of mankind and the moral obligations connected to the birth of entities with possible human-like characteristics.

Furthermore, the possibility of using SEMs for reproductive purposes goes beyond simple investigation. SEMs could be models for understanding infertility; nonetheless, the idea of using synthetic embryos to produce live human kids raises moral questions. Main concerns are the probable commodification of human life, the safety and efficiency of using SEM-derived gametes for reproduction, and potentially socio-legal consequences [65]. Even just for research purposes, the creation of human embryos lacking natural fertilization challenges current bioethical paradigms and calls for clear regulatory control to ensure responsible scientific inquiry [67].

### Public Perception and Societal Impact

A complex interaction of ethical, cultural, and scientific elements shapes the public view of SEM research. Though there is general agreement on the possible advantages of SEMs for improving medical knowledge and treating diseases, ethical questions surround the creation of embryo-like creatures in laboratory environments [39]. The way SEMs are portrayed in the media as “synthetic embryos” could raise questions about possible usage and dehumanization, therefore aggravating these fears [59].

Establishing trust and ensuring legislative systems fit social conventions calls for public participation in debates on SEM research. Clear communication of SEM research’s goals, constraints, and ethical issues helps to dispel misunderstandings and promote educated public discussion [61]. Including ethicists, legislators, and community leaders among the stakeholders involved in developing rules guarantees that SEM research follows society’s expectations and values [63].

Synthetic embryo models promise to solve critical medical problems and improve our knowledge of human development. But its use generates complex moral and legal conundrums that demand careful thought. Establishing clear guidelines for SEM testing, clarifying the ethical and legal consequences of these models, and involving the public in debates on their possible applications help one to do SEM research responsibly and ethically. By balancing ethical responsibilities with scientific advancement, we can use SEMs to maintain society’s standards while improving human health.

## Improving Ethical Communication on Synthetic Embryo Models

Synthetic embryo models (SEMs) have created various moral conundrums as well as legal ones. These models question the accepted ethical and legal systems controlling human embryo research even while they provide revolutionary opportunities for disease study and regenerative medicine. Examining case studies including the International Society for Stem Cell Research (ISSCR) recommendations and national limitations in conjunction with ethical frameworks such “developmental potential” and “specific criteria” helps one to have a thorough understanding of these ethical issues. This essay looks at these qualities to strengthen the ethical discussion around SEMs.

### Case Studies in Regulatory and Ethical Concerns

Stem cell and embryo research ethical and legal rules have been established in great part because of the ISSCR. Eliminating the strict 14-day limit for human embryo culture and encouraging case-by-case assessments anchored in scientific and ethical concerns helped the 2021 ISSCR guidelines to have a more flexible framework [68]. This shift shows growing awareness of the fact that SEMs—which copy specific aspects of embryonic development—need special attention apart from standard human embryos. While stressing the need of ongoing ethical reflection especially when SEMs blur the lines between artificial models and real human embryos, the ISSCR criteria support responsible research.

Different countries have put different policies in place for SEM research, reflecting cultural, religious, and legal differences in opinions about the moral standing of embryos. With strict restrictions on embryo research, the Human Fertilization and Embryology Authority (HFEA) in the United Kingdom follows the 14-day guideline [69]. Though synthetic models are not directly subject to these criteria, debates about their administration and suitable monitoring structure continue. With funding organizations like the National Institutes of Health (NIH), which forbids federal financing for human embryo research [70], monitoring SEMs is mostly assigned to institutional review boards (IRBs), hence there is a lack of federal control especially over SEMs in the United States. Japan has adopted a sensible but forward-looking strategy, allowing extended embryo development under particular ethical constraints but maintaining restrictions on implantation [71]. The different national strategies emphasize the need for world agreement while respecting cultural and ethical variety.

### Ethical Frameworks for Evaluating SEMs

Mostly ethical debates regarding SEMs center on their “developmental potential.” This idea separates those devoid of such potential (e.g., SEMs made to duplicate particular stages without complete developmental capacity) from those able of evolving into viable organisms (e.g., natural embryos) (Green, 2020). Proponents argue that SEMs should not be given equal moral value as human embryos since they cannot mature into a completely evolved human being. Critics caution that the difference between models and viable embryos may blur as technology develops, so constant ethical review is needed [72, 73].

Another ethical perspective determines moral status by evaluating species-specific criteria. According to this point of view, moral assessment should be based not only on developmental possibilities but also on the existence of human genetic material and morphological resemblance to natural embryos [74]. Some ethicists argue that if a somatic embryonic model closely resembles a human embryo in structure and function, it should be controlled by corresponding ethical and legal systems. Some contend, on the other hand, that SEMs should be ethically distinguished from human embryos as long as they do not satisfy basic biological criteria for personality or sentience [75].

In conclusion, the ethical debate on SEMs has to advance in line with scientific advances using advanced points of view that take new ethical frameworks into account as well as regulatory precedents. Insightful case examples showing several approaches for the control of stem cell research are provided by the ISSCR guidelines and national policies. Concurrently, models such as “developmental potential” and “species-specific criteria” offer methodical approaches for assessing SEM moral situation. Including these elements into an improved ethical debate would encourage responsible innovation and help to solve social issues in the direction of synthetic embryo research.

## Current Limitations of Stem Cell-Based Embryo Models

Stem cell-based embryo models have great promises to revolutionize the field of human embryogenesis; nonetheless, important issues must be resolved before their general use. Especially prone to failure, pre-implantation models such as blastoids cannot recreate early developmental phases, including cleavage and morula stages. Although blastoids can replicate some features of trophectoderm specification, their control in blastocysts—especially about hypoblast specification—is not precisely reflected. Furthermore, improperly adjusted current treatments may cause off-target cells to diverge from the usual developmental route to the surface. Moreover, ethical issues prevent blastoids from being put into a host uterus for functional evaluation; their developmental ability is limited, particularly in models of mice and primates. A difficulty with post-implantation models is the notable variation in cell type proportions and developmental outcomes, which can vary significantly between trials depending on the model, technique, and starting conditions. Problems cover genetic abnormalities in naive pluripotent stem cells and challenges in precisely counting cells in various stem cell cultures, aggravating variability because of these variations in medium conditions and difficulty normalizing data. Using cooperative efforts to exchange techniques and enhance the repeatability of these models could resolve these challenges in the future [12].

### Challenges of Synthetic Embryo Models

Synthetic embryo models have transforming power, but they also face many major challenges that prevent their broad application in developmental biology, disease modeling, and regenerative medicine. These problems cover technical, biological, ethical, and legal elements, therefore stressing the need of continuous improvement and creativity in this field.

### Technical and Biological Barriers

A key technological obstacle with synthetic embryo models is the lack of vascularization. A healthy circulatory system is required throughout proper embryonic development for the exchange of nutrients and oxygen and for waste removal. Modern models, particularly post-implantation structures, sometimes lack the ability to create circulatory networks, therefore limiting their viability and growth outside of the first embryonic phases. Inadequate blood flow limits the efficacy of these models in investigating complex organogenesis and fetal development by making advanced phases of embryogenesis less faithfully replicated [76].

The stability and repeatability of synthetic embryo models provide still another challenge. Stem cell-derived models show variation in their abilities for self-organization and advancement through embryonic stages even in carefully controlled laboratory environments. Standardizing this heterogeneity complicates data comparability across numerous studies and makes translational applications difficult. Often, they lack essential extraembryonic components like the placenta and yolk sac, which are essential for nutrient exchange and signaling during development, synthetic models The lack of these supporting systems compromises the physiological relevance of these models even further [8].

### Ethical and Legal Challenges

Particularly as synthetic embryo models advance and progressively replicate later stages of human life, their creation and use raise serious ethical questions. Given their increasing resemblance to natural organs, especially, the moral and legal status of these models presents a major ethical question. Different countries have current policies that vary; some follow strict guidelines on the creation and research on synthetic models. The “14-day rule,” which limits human embryo research to the initial two weeks of development, has historically delineated ethical bounds; yet, its relevance to synthetic models continues to be a subject of vigorous controversy [62].

The challenges involved in functional testing raise still another ethical issue. As researchers advance synthetic embryo models along the developmental continuum, evaluating their viability and usefulness in implantation-like contexts presents intricate ethical and scientific dilemmas. Although these investigations may yield revolutionary insights into early human development and their therapeutic uses, they also pose a risk of conflating experimental models with real embryos, hence requiring meticulous ethical supervision and regulatory structures [61].

### Potential Techniques for Reducing Restraints

To overcome these restrictions, a multidisciplinary strategy that integrates advancements in bioengineering, stem cell biology, and computational modeling is essential. Integrating vascularization, enhancing repeatability, and developing bioengineered support systems could substantially augment the functionality of synthetic embryo models. Moreover, explicit ethical rules and regulatory frameworks must develop concurrently with scientific progress to guarantee responsible research while optimizing the capabilities of these models in medicine and biology.

### Public Contribution to Synthetic Embryo Research

Addressing the ethical, social, and scientific consequences of synthetic embryo research depends on significant public involvement as it develops. Although some debates on public participation are underway, thorough plans involving the public in a deeper conversation on the hazards, advantages, and social effects of these technologies are still much needed. Improving knowledge and understanding synthetic embryo research depends critically on public involvement programs including citizen science projects and instructional efforts.

Efforts at citizen science offer a unique chance for public involvement in data collecting, analysis, and project design. Including laypersons into the research process helps scientists to address issues and misunderstandings about synthetic biology at the same time and promote a more inclusive and open debate. These projects could be especially helpful in talking about the ethical limits of synthetic embryos since they give people from many backgrounds a platform to express their ideas and help to influence decisions [77].

Furthermore, educational initiatives work well as a tool for raising public awareness and promoting public conversation. Such initiatives could include internet tools, public talks, and workshops meant to provide easily available clarifications of synthetic embryo research. These sites could clarify important questions about the differences between synthetic and traditional embryos, the possible therapeutic benefits, and the ethical conundrums related to these technologies. Particularly in complex and ethically sensitive fields like human embryo modeling [78], public education clarifies scientific ideas and helps people to develop educated opinions on topics that greatly influence society. The spread and conversation of science outside of the scientific community depends on public education to be facilitated.

### Regional Variations and an International Regulatory Framework

Although the International Society for Stem Cell Research (ISSCR) recommendations offer a useful framework for synthetic embryo research, the regulatory environment differs greatly depending on where one is working. Although it ignores the major issue of regional variations in synthetic embryo model policy, particularly between Asia and Europe, the evaluation mostly addresses the ISSCR criteria. These variations have significant consequences for global synthetic biology cooperation as well as for ethical behavior in research.

Strict ethical guidelines controlling human embryo research help to set rules on synthetic embryo research in Europe. The rules of the European Union on human stem cell research often stress the preservation of human dignity, which translates into more limited guidelines on the development and modification of synthetic embryos. Following the “14-day rule” that restricts the culture of human embryos for scientific uses outside two weeks, many European nations [79]. These laws guarantee that synthetic embryo research stays inside moral limits, but they could also restrict the extent of experimentation and model development.

On synthetic embryo research, numerous Asian nations—including China and Japan—have embraced more permissive legal policies. These nations are often more receptive to the application of modern biotechnologies, therefore enabling faster innovation and less constraints on the production and modification of synthetic embryos. For instance, China’s regulations have been somewhat liberal, which has spurred fast developments in synthetic embryo models’ application and stem cell research [80]. This adaptability has, however, also sparked questions over the absence of robust ethical supervision and recommendations for more thorough control to guarantee responsible research practices [81].

The differences in regulations between countries provide difficulties for global cooperation and the uniformity of moral guidelines in synthetic embryo research. Global regulatory agencies and local governments must create shared policies that respect cultural and ethical variances while advancing responsible research if they are to solve these challenges. Encouragement of worldwide debates and best practices will assist in closing these gaps and guarantee that synthetic embryo research proceeds in a morally sound, scientifically accurate, and socially acceptable manner all around [82].

## Future Directions

Future studies have to balance moral responsibilities with the advancement of science. Advances in bioengineering, better cellular differentiation methods, and artificial intelligence integration to replicate embryonic development will expand the use of SEM. Regulatory systems must change concurrently with new technologies to guarantee suitable research techniques. The rapid advances in stem cell research and synthetic embryo models (SEMs) have greatly improved our knowledge of early development, disease modeling, and regenerative medicine. As these technologies develop, some important areas need more research to realize their potential and handle ethical, technological, and legal issues. Five main directions in this field can be distinguished: augmenting model complexity, advancing translational applications, including computational modeling and artificial intelligence (AI), improving ethical and regulatory frameworks, and encouraging public participation and policy development.

A primary difficulty in SEM research is raising the precision of these models to replicate natural embryogenesis faithfully. Modern scanning electron microscopes lack complete signaling links, cellular heterogeneity, and spatial structure in actual embryos, even if they can replicate critical developmental periods. Integrating different cell types, particularly extraembryonic tissues, should be the main emphasis of further studies to improve model survivability and self-organization. Modern bioengineering techniques, including 3D bioprinting and microfluidics, allow one to reproduce the microenvironment required for more physiologically realistic models. Furthermore, the efficiency of lineage-specific differentiation produced in SEMs will determine the formation of organoid-like structures with functional properties.

Besides fundamental developmental biology, SEMs have possible uses in translational medicine, covering drug development, illness modeling, and customized therapy. Future research with SEMs should mainly concentrate on congenital diseases, placental anomalies, and infertility. Furthermore, providing a fresh approach to precision medicine, using induced pluripotent stem cells (iPSCs) to create patient-specific stem cell-derived extracellular matrices (SEMs) could help provide tailored therapeutic intervention assessments. In regenerative medicine, SEM findings could help develop tissue engineering, organ regeneration, and cell-based therapy for degenerative diseases.

The complexity of embryonic development calls for sophisticated computational techniques to clarify complicated gene control networks and cellular interactions. Artificial intelligence and machine learning methods allow one to investigate extensive single-cell transcriptomics, proteomics, and epigenomics datasets to generate prediction models of cellular destiny decisions. In silico modeling, embryogenesis can also help experimental research by mimicking embryonic processes, spotting new regulating mechanisms, and enhancing culture conditions for stem cell-derived embryoids. Integrating artificial intelligence-driven automation into SEM studies should be the main emphasis of future studies to improve repeatability, speed discovery, and real-time experimental parameter modification.

The ethical and legal terrain has to change with the evolution of SEM complexity. The unique qualities of SEMs could call for a review of present limits, particularly the 14-day limit on human embryo research. The main focus of ethical debates should be whether undeveloped SEMs should be limited to normal embryos. Explicit international rules are required to guarantee moral research methods, stop exploitation, and set monitoring systems. Future discussions should consider synthetic gametes and their possible use in reproductive technology.

General public opinion of SEM research significantly affects legislative decisions and financing choices. Encouragement of educated debates on the social consequences of SEM research calls for honest communication and active public participation, as well as patient advocacy groups, ethicists, legislators, and public servants. Future projects should concentrate on media involvement, educational programs, and multidisciplinary forums to close the knowledge difference between public opinion and scientific discoveries. Furthermore, cooperative projects between academic institutions and regulatory authorities should prioritize creating flexible rules reflecting the changing scene of stem cell research and SEM. Stem cells and artificial embryo models have great potential to improve our knowledge of human development and enable future biomedical uses. Thanks, of course. By simplifying models, improving translational applications, integrating computational approaches, addressing ethical issues, and encouraging public debate, the discipline may surpass upcoming problems and guarantee responsible and significant scientific advancement. Significant expenditures in multidisciplinary cooperation, regulatory systems, and technological improvements will be essential if we entirely use SEMs in the following decades.

## Conclusion

Synthetic embryo models represent a revolutionary breakthrough in developmental biology since they offer strong tools to dissect early human development and disease pathways. Their ability to replicate fundamental embryonic events presents unmatched opportunities for research on congenital disorders, regenerative medicine advancement, and drug discovery systems enhancement. Still, these advances raise serious ethical and legal questions that must be addressed to ensure responsible scientific progress. Dealing with the complexities of SEM research will depend on well-defined standards, encouragement of international collaboration, and involvement of various stakeholders—such as scientists, ethicists, and legislators. By encouraging multidisciplinary dialogue and harmonizing innovation with ethical responsibility, the field may fully exploit the promise of synthetic embryo models while safeguarding moral principles and society. The continuous development of this transforming technology depends on constant efforts to improve experimental approaches, including computer instruments, and apply strict control systems.

## Data Availability

The paper and its supplementary information contain all the data supporting this review article.
